# Machine learning models for net photosynthetic rate prediction using poplar leaf phenotype data

**DOI:** 10.1371/journal.pone.0228645

**Published:** 2020-02-11

**Authors:** Xiao-Yu Zhang, Ziyuan Huang, Xuehui Su, Andrew Siu, Yuepeng Song, Deqiang Zhang, Qing Fang

**Affiliations:** 1 College of Science, Beijing Forestry University, Beijing, P. R. China; 2 Data Science, Harrisburg University of Science and Technology, Harrisburg, PA, United States of America; 3 Jiaozuo Academy of Agriculture and Forestry Sciences, Jiaozuo, P. R. China; 4 Amgen Inc., Thousand Oaks, CA, United States of America; 5 College of Biological Sciences and Technology, Beijing Forestry University, Beijing, P. R. China; 6 Faculty of Science, Yamagata University, Yamagata, Japan; Newcastle University, UNITED KINGDOM

## Abstract

**Background:**

As an essential component in reducing anthropogenic CO_2_ emissions to the atmosphere, tree planting is the key to keeping carbon dioxide emissions under control. In 1992, the United Nations agreed to take action at the Earth Summit to stabilize and reduce net zero global anthropogenic CO_2_ emissions. Tree planting was identified as an effective method to offset CO_2_ emissions. A high net photosynthetic rate (Pn) with fast-growing trees could efficiently fulfill the goal of CO_2_ emission reduction. Net photosynthetic rate model can provide refernece for plant’s stability of photosynthesis productivity.

**Methods and results:**

Using leaf phenotype data to predict the Pn can help effectively guide tree planting policies to offset CO_2_ release into the atmosphere. Tree planting has been proposed as one climate change solution. One of the most popular trees to plant are poplars. This study used a *Populus simonii* (*P*. *simonii*) dataset collected from 23 artificial forests in northern China. The samples represent almost the entire geographic distribution of *P*. *simonii*. The geographic locations of these *P*. *simonii* trees cover most of the major provinces of northern China. The northwestern point reaches (36°30’N, 98°09’E). The northeastern point reaches (40°91’N, 115°83’E). The southwestern point reaches (32°31’N, 108°90’E). The southeastern point reaches (34°39’N, 113°74’E). The collected data on leaf phenotypic traits are sparse, noisy, and highly correlated. The photosynthetic rate data are nonnormal and skewed. Many machine learning algorithms can produce reasonably accurate predictions despite these data issues. Influential outliers are removed to allow an accurate and precise prediction, and cluster analysis is implemented as part of a data exploratory analysis to investigate further details in the dataset. We select four regression methods, extreme gradient boosting (XGBoost), support vector machine (SVM), random forest (RF) and generalized additive model (GAM), which are suitable to use on the dataset given in this study. Cross-validation and regularization mechanisms are implemented in the XGBoost, SVM, RF, and GAM algorithms to ensure the validity of the outputs.

**Conclusions:**

The best-performing approach is XGBoost, which generates a net photosynthetic rate prediction that has a 0.77 correlation with the actual rates. Moreover, the root mean square error (RMSE) is 2.57, which is approximately 35 percent smaller than the standard deviation of 3.97. The other metrics, i.e., the MAE, R^2^, and the min-max accuracy are 1.12, 0.60, and 0.93, respectively. This study demonstrates the ability of machine learning models to use noisy leaf phenotype data to predict the net photosynthetic rate with significant accuracy. Most net photosynthetic rate prediction studies are conducted on herbaceous plants. The net photosynthetic rate prediction of *P*. *simonii*, a kind of woody plant, illustrates significant guidance for plant science or environmental science regarding the predictive relationship between leaf phenotypic characteristics and the Pn for woody plants in northern China.

## Introduction

In 2018, the United Nations’ Intergovernmental Panel on Climate Change (IPCC) published a special report called Global Warming of 1.5°C to warn the world that countries must reduce their greenhouse gas (GHG) emissions as quickly as possible to avoid adverse consequences due to climate change [1]. Human activities influence the global mean surface temperature (GMST). The estimated temperature impact by human activities is an approximate 1.0°C increase compared to the pre-industrial temperature. Maintaining or reducing the net zero global anthropogenic carbon dioxide (CO_2_) emission could stop anthropogenic global warming in the future [1].

As an essential component in reducing anthropogenic CO_2_ emissions to the atmosphere, tree planting is the key to keeping carbon dioxide emissions under control. In 1992, the United Nations agreed to take action at the Earth Summit to stabilize and reduce the net zero global anthropogenic CO_2_ emissions. Tree planting was defined as one of the more effective methods to offset CO_2_ emissions [2]. A high net photosynthetic rate (Pn) with fast-growing trees could efficiently fulfill the goal of CO_2_ emission reduction.

*P*. *simonii* is one of the fastest growing trees in the world and has the potential to get anthropogenic CO_2_ emissions under control quickly and effectively. Net photosynthetic rate represents the level of plant photosynthesis. The research of *P*. *simonii*’s net photosynthetic rate prediction is of practical significance in determining carbon fixation and promoting plant growth and development [3]. The Pn is the most important index in evaluating CO_2_ emission reduction, which is discussed in this paper. Accurate prediction of the Pn could be an authoritative reference for analyzing and evaluating carbon sequestration. However, measuring photosynthesis data is a challenge impacted by heterogenetic environmental parameters. For example, light intensity, CO_2_ concentration, water availability, and temperature are the key factors that affect the Pn. When any of these factors become a limiting factor, this limiting factor masks the effects of the other parameters [4]. Due to the limiting factor effect and environmental variations, a researcher must bring many devices into a forest to measure environmental variables such as the Pn, which is costly and logistically difficult. We hypothesize that it is possible to use leaf phenotype data to accurately predict the Pn so that forest researchers can evaluate and analyze Pn exactly, effectively, and economically.

Early photosynthesis predictive models tended to be simple and straightforward. Long and Incoll (1975) introduced a leaf photosynthetic rate model that used a variant of the ^14^CO_2_ method to predict the photosynthetic rate of *Spartina townsendii* (*S. townsendii*) based on their 2-gas system for photosynthetic rate measurement and sampling strategy [5]. In 1991, Ögren published a paper using a multiple regression model to study the photoinhibition of photosynthesis for willow (*Salix sp*.) leaves with the ratio of variables to the maximal chlorophyll fluorescence [6]. This research did address the problem that Long and Incoll experienced in 1975: there were multiple independent variables with changing environmental conditions that strongly impacted the photosynthesis prediction results.

Later investigations regarding photosynthesis were conducted in controlled environments with limited environmental and geographical influences and implemented more sophisticated approaches to create more robust and reliable predictions. Machine learning was one of the most popular methods for analyzing photosynthesis data and making predictions. Lü et al. (2017) used hyperspectral data of wheat flag leaves to predict the Pn with three machine learning models: 1) quadratic polynomial stepwise regression (QPSR), 2) partial least squares regression (PLSR), and 3) a backpropagation neural network (BPNN). These methods were able to generate root mean square error (RMSE) values of 0.71, 0.86, and 0.78 with residual predictive deviation (RPD) values of 2.9, 2.4, and 2.6, respectively [7]. Zhang et al. (2019) also used hyperspectral data to predict winter wheat leaves’ max net photosynthetic rate (A_max_) using partial least squares (PLS), a support vector machine (SVM), multivariate linear regression (MLR) and an artificial neural network (ANN) [8]. Since then, the machine learning and neural networks for hyperspectral data are the contemporary approaches dominating in Pn prediction with relatively reasonable R^2^ and RMSE values.

Leaf shape characteristics are associated with Pn predictions. Ci et al. (2015) published a paper concluding that twenty-six leaf-shape-related methylation markers are significantly associated with photosynthetic characteristics [9]. In March 2019, Zhang et al. invented an iteration-stopping criterion for gradient boosting machine (GBM) models to reduce the impact of overfitting as a preliminary study for Pn prediction and used 235 *P*. *simonii* leaf phenotype and photosynthesis data points [10]. Since most of the photosynthesis studies in this domain are focused on herbaceous plants, Pn prediction using *P*. *simonii* leaf phenotype data can be a particularly useful reference for woody plant studies in northern China.

## Materials

### Experimental design and data acquisition conditions

To obtain the maximum Pn data without being impacted by environmental and geographical variations, 519 *P*. *simonii* root cuttings were brought to grow in a clonal arboretum located in Guan County, Shandong Province, China (36°23’N, 115°47’E), in a controlled environment. The samples represent almost the entire geographic distribution of *P*. *simonii* (9 provinces, 17 cities and counties: Chicheng County and Zhangjiakou City from Hebei, Fu County, Linyou County, Langao County, Luochuan County and Gaoling County from Shaanxi, Huzhu County, Xinghai County, Dulan County and Menyuan County from Qinghai, Song County and Yichuan County from Henan, Zhongning County from Ningxia, Baotou City from Inner Mongolia, Ningwu County from Shanxi, Taoranting Park in Beijing). We have obtained the work permit of the experimental base of *P*. *simonii* euphratica germplasm resources from Guan County Governmet. The data collection time was set between 9:00 AM and 11:00 AM. During this period, the Pn peaks during a day. The whole experiment was finished by the National Engineering Laboratory for Tree Breeding, College of Biological Sciences and Technology, Beijing Forestry University.

When collecting leaf phenotype and photosynthetic data, the portable laser leaf area meter (CI-202) and the portable photosynthesis system (LI-6400XT) measured the top four to six leaves three times from the stem top and then took the average as each individual tree’s leaf shape data. The photosynthetic data collection parameters needed to meet the following conditions: 1) the photosynthetic photon flux density (PPFD) was set to 1600 *μmol* ⋅ *m*^−2^*s*^−1^, 2) the CO_2_ concentration was set to 400 *μmol*^−1^, and 3) the Pn stability level was reached at ±0.1. These leaf phenotypic and photosynthetic data collection conditions ensured the robustness, reliability, and stability of the collected data (refer to Supplementary Data of doi.org/10.1093/jxb/erv485 at Journal of Experimental Botany (online) https://academic.oup.com/jxb).

### Dataset

The 519*P*. *simonii* samples originally came from 23 forests in northern China. These poplar trees represent the majority of the characteristics of the artificial poplar forests planted in northern China. The dataset collected using CI-202 and LI-6400XT systems includes the area, length, width, perimeter, ratio, factor, and Pn. There are six predictors in this study: the area, length, width, perimeter, ratio, and factor. Area stands for the leaf area and measures how large a leaf is. Length stands for leaf length and measures the maximum length of a leaf. Width stands for leaf width and measures the maximum width of a leaf. Perimeter stands for leaf perimeter and measures the distance around a leaf. The ratio represents the aspect ratio. The aspect ratio is calculated using the leaf length divided by the leaf width. The factor represents the shape factor. The shape factor is calculated as $4\pi \frac{\text{area}}{{\text{perimeter}}^{2}}$. The last variable is the Pn, which is the response variable in this study. The Pn is the net photosynthetic rate and is the difference between the CO_2_ update and CO_2_ release. The Pn is a crucial index to measure precisely how much the photosynthesis process absorbs CO_2_.

## Methods

### Exploratory analysis of the data

**Fig 1:**
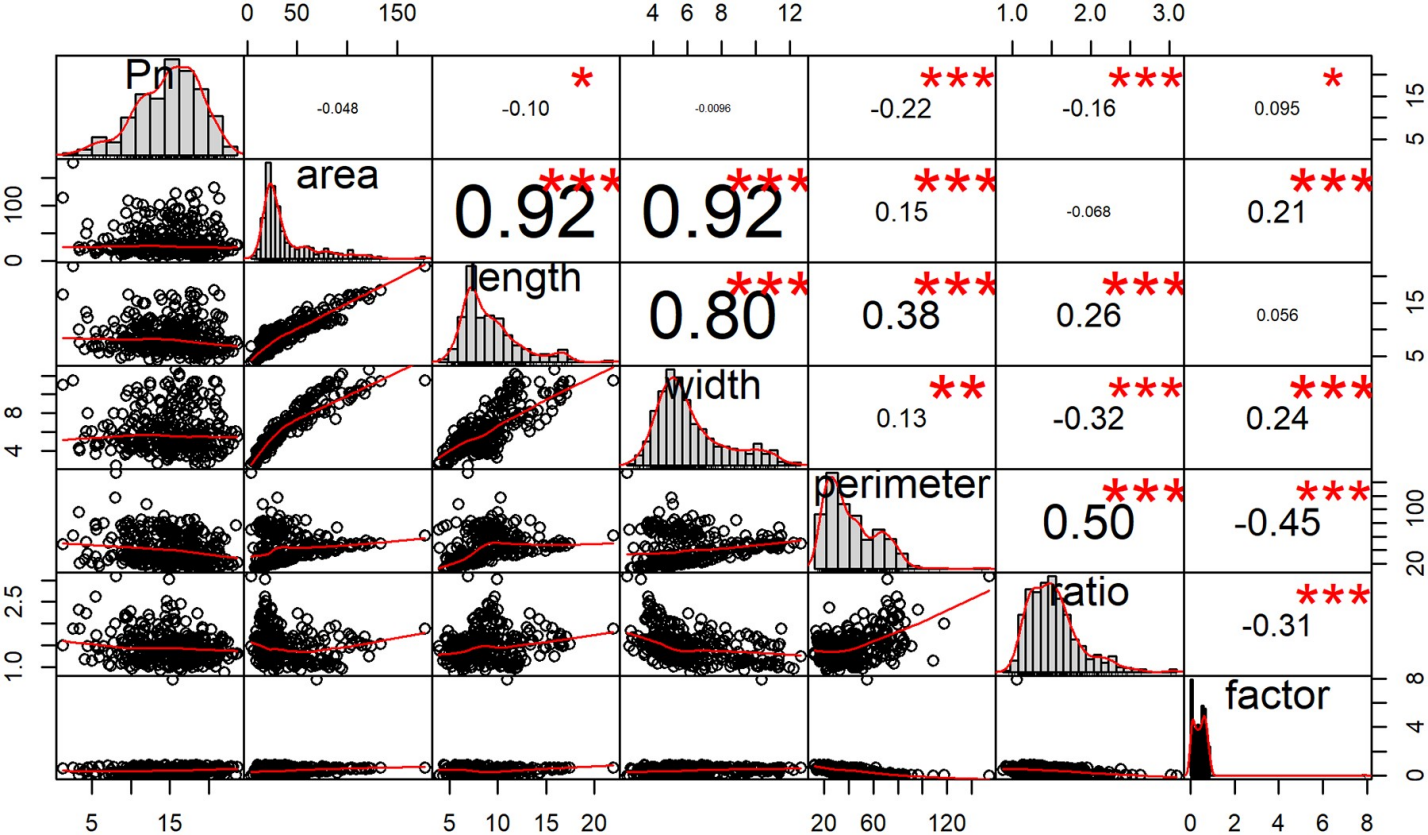
Dataset correlation. The correlation patterns of Pn and six predictors with area, length, width, perimeter, ratio, factor (R package PerformanceAnalytics).

The net photosynthetic rate (Pn) reflects the important ability of storing energy and organics for various plants. The purpose of this paper is to study the relationships between net photosynthetic rate and leaf phenotypic characteristics. Therefore we focus on how we could use a machine learning model to predict Pn when the collected data is noisy, skewed and highly correlated with influential outliers. We note that such patterns reflect the characteristics of datasets in the big data era. The 3Vs (volume, variety, and velocity) always break assumptions in the white-box modeling process that cause the unreliability of many statistical models. However, black-box modeling is capable of coping with noisy, skewed, and highly correlated datasets because most black-box modeling algorithms do not make distribution assumptions. The following listed patterns of the dataset in this study (Fig 1 and Tables 1–3).

**Table 1:** Multivariate normality tests (R package MVN).

Test	Statistic	p-value	Result
Mardia’s Skewness	32276.54	0.00	NO
Mardia’s Kurtosis	432.27	0.00	NO
MVN	NA	NA	NO

**Table 2:** Univariate normality tests (R package MVN).

Test	Variable	Statistic	p-value	Normality
Shapiro-Wilk	Pn	0.98	<0.001	NO
Shapiro-Wilk	area	0.78	<0.001	NO
Shapiro-Wilk	length	0.89	<0.001	NO
Shapiro-Wilk	width	0.91	<0.001	NO
Shapiro-Wilk	perimeter	0.91	<0.001	NO
Shapiro-Wilk	ratio	0.92	<0.001	NO
Shapiro-Wilk	factor	0.50	<0.001	NO

**Table 3:** Descriptives with n = 519 (R package MVN).

	Mean	Median	Min	Max	25th P	75th P	Skewness	Kurtosis
Pn	14.57	15.20	1.32	23.59	11.66	17.76	−0.52	−0.05
area	35.83	24.33	3.91	177.95	18.76	43.02	1.87	3.63
length	8.58	7.76	3.88	21.96	6.41	9.96	1.28	1.59
width	6.02	5.41	2.44	12.66	4.52	6.98	1.03	0.32
perimeter	41.36	34.38	12.41	154.02	23.51	56.88	0.92	0.75
ratio	1.47	1.42	0.88	3.09	1.23	1.63	1.19	2.00
factor	0.42	0.41	0.00	7.90	0.14	0.63	10.95	193.68

According to Fig 1 and Tables 1–3, the dataset follows neither a multivariate nor a univariate normal distribution. Therefore, the variables in the dataset are either left or right skewed and highly correlated. Thus, appropriate data preprocessing mechanisms are needed. Then, based on the results after data preprocessing, the appropriate predictive methods should be selected to make valid and accurate Pn predictions.

#### Data preprocessing

**Fig 2:**
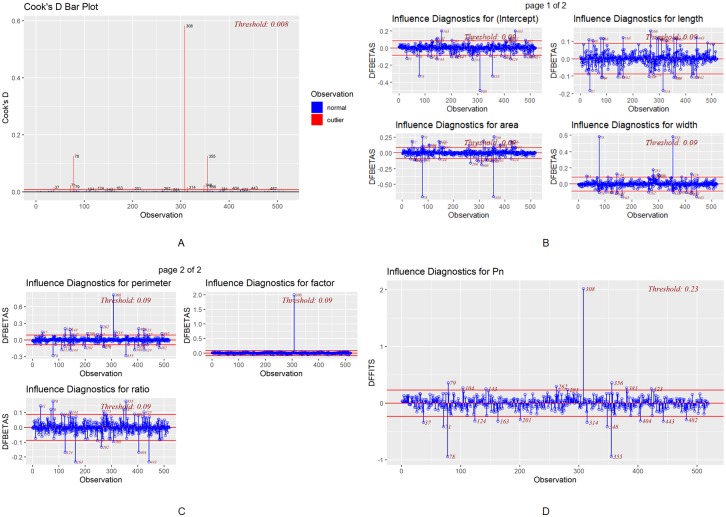
Influence diagnostics. (A) Cook’s distance bar plot with threshold 0.008, (B) DFBETAs panels for intercept, area, length and width with threshold 0.09, (C) DFBETAs panels for perimeter, ratio and factor with threshold 0.09, (D) DFFITS plot for Pn with threshold 0.23 (R package olsrr).

To conduct appropriate data preprocessing, we should first identify outliers. By conducting Cook’s Distance, the DFBETAs, and the DFFITS, we can observe a few outliers. There are a few extreme values show in the bar plot of Cook’s distance. For example, the 78th, 308th, and 355th rows are apparent outliers that deviate significantly from the mean (refer to Fig 2A). In addition, for each variable, multiple outliers could significantly impact the overall analysis. For the length variable, the 37th, 163rd, 266th, and 314th rows are influential data points that could affect the analysis results (refer to Fig 2B). Moreover, for the perimeter variable, the 28th, 144th, 262nd, and 308th rows are influential outliers that could impact the perimeter analysis (refer to Fig 2C).

To achieve accurate and precise predictions, the influential outliers are removed because extreme values may mislead the analysis results. Cook’s distance is chosen in this study to remove outliers due to its reliability for multivariate data. The outlier identification threshold is set to three times the mean, which is a typical threshold for targetting outliers.

**Fig 3:**
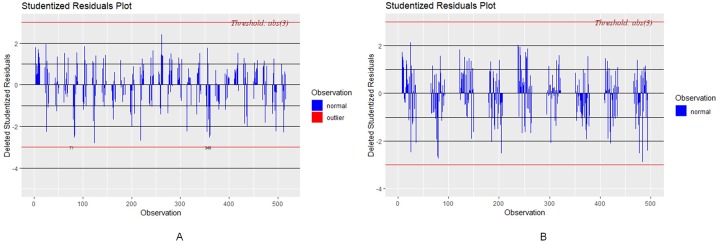
Studentized residuals plots. (A) Before outlier removal with threshold abs(3), (B) After outlier removal with threshold abs(3) (R package olsrr).

Based on Cook’s distance, the influential outliers were removed from the dataset (refer to Fig 3A and 3B). According to the Studentized residuals plot before and after outlier removal, no other outliers were identified after the initial outliers were removed.

#### Cluster analysis

Due to different geographical location and growth environment, *P*. *simonii* may have different characteristics so that they can be divided into different subpopulations. In our research, we utilize partitioning around medoids (PAM) clustering to classify these *P*. *simonii* individuals to study the relationship between geographical distribution and *P*. *simonii*’s leaf phenotypic traits and photosynthetic characteristics.

A cluster analysis was implemented as part of the data exploration step to investigate additional details in the dataset. The first step was to define the optimal number of clusters. Gower’s distance was used to measure the distance between different data points. Then, partitioning around medoids (PAM) was implemented to define medoids in the center of each cluster. The goal of PAM is to minimize the distance within a cluster and maximize the distance between different clusters [11]. After that, the silhouette width from the PAM algorithm reveals the optimal number of clusters for the given dataset and how strong the clustering structure is.

**Fig 4:**
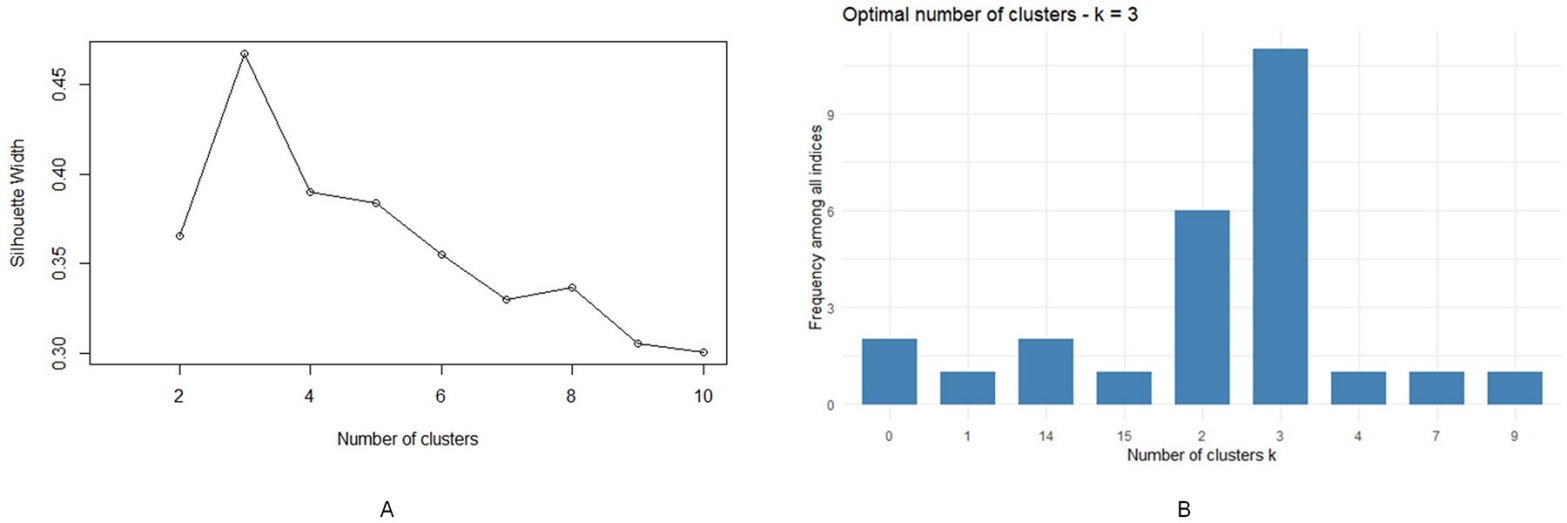
PAM results. (A) Silhouette width diagram, (B) Frequency among all indices with optimal number of clusters k = 3 (R packages graphics and NbClust).

Based on the silhouette width diagram (refer to Fig 4A), 3 is the optimal number of clusters with a silhouette width score of 0.47, which means that a clustering structure does exist, but further analysis is required to ensure that the cluster number is valid and optimized.

Thus, we implemented k-means clustering by means of the Euclidean distance with a comparison of a dozen of indices: KL, CH, Hartigan, CCC, Scott, Marriot, TrCovW, TraceW, Friedman, Rubin, Cindex, and many others. A cluster number of 3 was identified as optimal when compared to other potential numbers of clustered and was proposed 11 times among all the indices (refer to Fig 4B).

**Fig 5:**
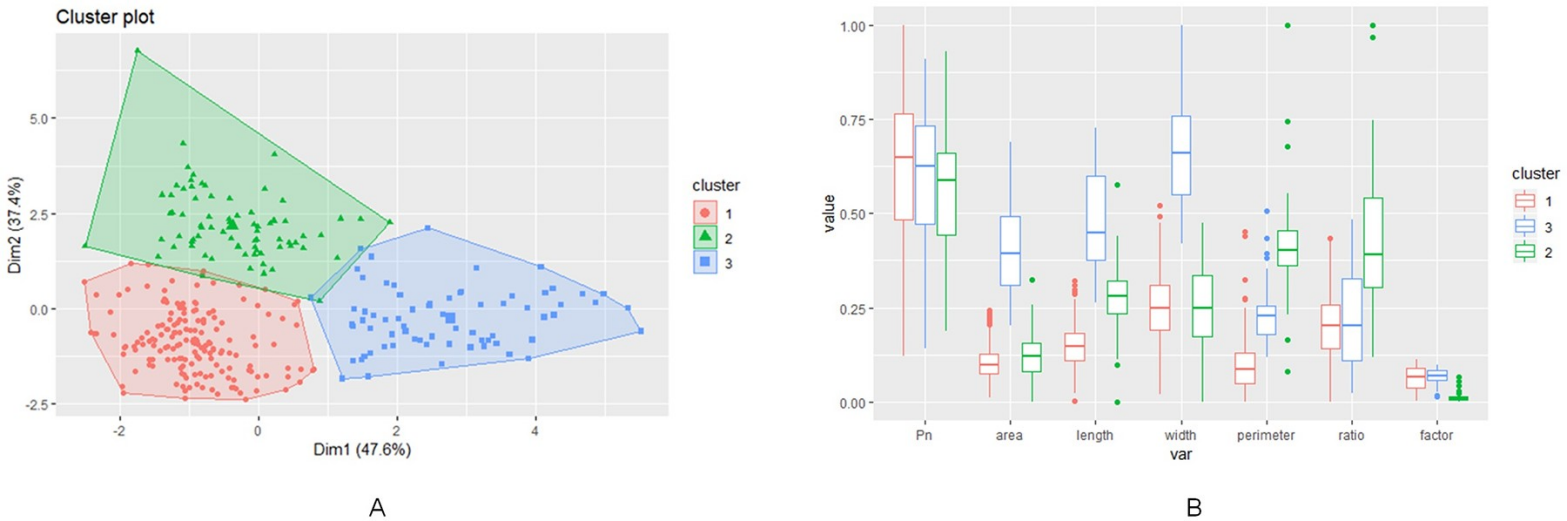
Clustering of the leaf phenotypic traits. (A) Cluster plot where colors red, green and blue correspond to cluster 1, 2 and 3, respectively, (B) Box plot for Pn, area, length, width, perimeter, ratio and factor (R packages factoextra and ggplot2).

Based on the calculated optimal number of clusters using the k-means method, the phenotype data can be plotted into three clusters (refer to Fig 5A).

We can observe a few patterns of the leaf phenotype data based on the clustering data within the normalized range from 0 to 1. The smaller the perimeter is, the higher the Pn. The lower the leaf shape ratio is, the more elevated the Pn. Regarding the leaf shape factor, the higher the leaf shape factor is, the higher the Pn. Therefore, the leaf area, length, and width do not have apparent patterns associated with Pn. However, there are some observable patterns between area, length, width, and perimeter. Cluster 1’s area, length, and width are much smaller than those of cluster 3, but cluster 1’s perimeter is the highest among the 3 clusters on average. Cluster 3’s area, length, and width are the highest among the 3 clusters, but the perimeter is much smaller than that of cluster 2. This finding can be interpreted as cluster 2’s leaves always having a smaller area, length, and width, but its leaf margin is still the most complicated among the 3 clusters. A typical leaf margin in cluster 2 may be much more lobed, wavy, spiny, or toothed than leaves in the other 2 clusters. The leaf margins of cluster 1 and cluster 3 are relatively smooth and straightforward (refer to Fig 5B).

**Fig 6:**
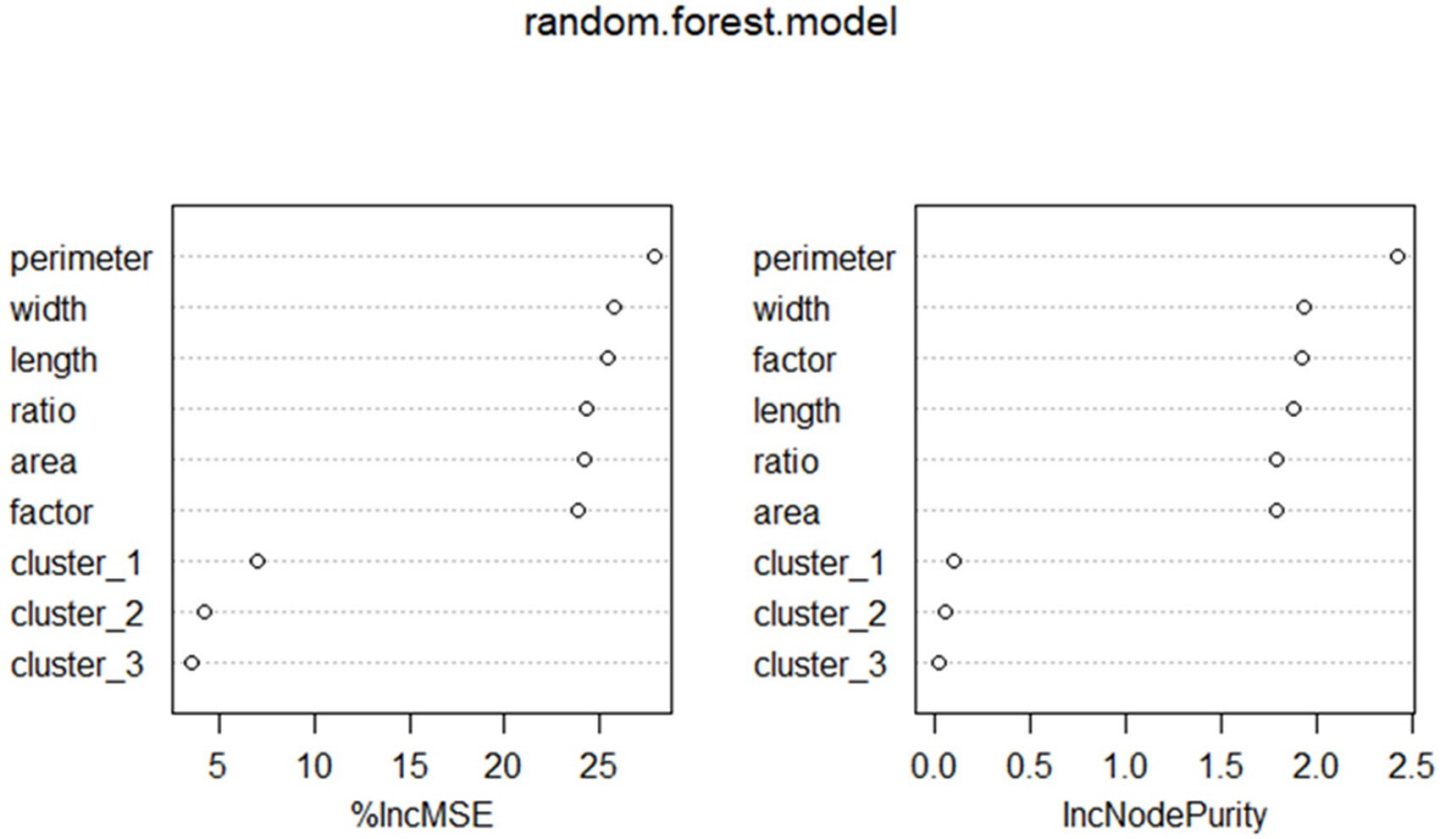
Results for random forest model. The left plot is variable importance plot with horizontal axis as %IncMSE, and the right one is variable importance plot with horizontal axis as IncNodePurity (R package graphics).

The purpose of the cluster analysis is to decide if multiple predictive models are needed or not. As a result, we do not have enough evidence to suggest statistically that different leaf phenotypic patterns yield different levels of Pn. Furthermore, the leaf phenotypic clustering characteristics’ influence on the Pn is minimal according to a random forest variable importance analysis (refer to Fig 6), which means that the clusters have a limited contribution to Pn prediction. We also conducted both analysis of variance (ANOVA) and Tukey’s honest significant differences (TukeyHSDs) to investigate whether different clusters generate different Pn levels. The results show that the ANOVA F-test for different leaf clusters is significant with a p-value equal to 0.0064. This finding means, in general, that different leaf clusters yield different Pn levels. However, according to TukeyHSD pairwise group comparisons, the only significant Pn generation difference exists between cluster 1 and cluster 2. Pn generation between cluster 1 and cluster 2 is not significantly different. Therefore, Pn generation between cluster 2 and cluster 3 is also not significantly different. Consequently, there is no need to develop multiple predictive models for each individual cluster. A well-trained predictive model should be sufficient to make Pn predictions in this case.

### Evaluation metrics

We implemented a few measures to evaluate the performance of the predictive models between the actual Pn and predicted Pn. The criteria used in this paper are the correlation (COR), root mean square error (RMSE), mean absolute error (MAE), coefficient of determination (R^2^), and the min-max accuracy. Correlation means the mutual relationship between the actual Pn and predicted Pn. The range of correlation is between -1 and 1. A positive correlation means that if the actual Pn increases, then the predicted Pn also increases, and vice versa. A negative correlation implies that if the actual Pn increases, then the predicted Pn decreases, and vice versa. A correlation of 0 indicates that there is no relationship between the actual Pn and the predicted Pn. The RMSE measures the errors between actual Pn and predicted Pn. The lower the RMSE is, the better the model. The RMSE is particularly powerful when working with large error outliers and might be highly useful for the dataset used in this research. The MAE is similar to the RMSE. However, the MAE works effectively with evenly distributed errors. R^2^ measures the proportion of the variance between the actual Pn and the predicted Pn. It reveals how much the predicted Pn is proportionally associated with the actual Pn. The min-max accuracy takes the mean values of the minimum and maximum values between the actual Pn and the predicted Pn to measure how close the predicted values are to the actual Pn values. The min-max accuracy ranges from 0 to 1. A min-max accuracy score of 1 means a perfect fit between the actual and predicted Pn values. The higher the min-max accuracy score is, the better the prediction. In addition, the RMSE is compared with the standard deviation (SD) to judge whether the predictive model performs better than using the mean for prediction.

**Formula of the correlation**

$$r_{xy}=\frac{\sum_{i=1}^{n}\left(x_{i}-\bar{x}\right)\left(y_{i}-\bar{y}\right)}{\sqrt{\sum_{i=1}^{n}{\left(x_{i}-\bar{x}\right)}^{2}{\left(y_{i}-\bar{y}\right)}^{2}}}$$ (1)

**Formula of the RMSE**

$$\text{RMSE}=\sqrt{\frac{\sum_{i=1}^{n}{\left(x_{i}-y_{i}\right)}^{2}}{n}}$$ (2)

**Formula of the MAE**

$$\text{MAE}=\frac{1}{n}\sum_{i=1}^{n}\left|x_{i}-y_{i}\right|$$ (3)

**Formula of R^2^**

$$\text{R}2=(1-\frac{\sqrt{\sum_{i=1}^{n}{\left(y_{i}-x_{i}\right)}^{2}}}{\sqrt{\sum_{i=1}^{n}{\left(y_{i}-\bar{y}\right)}^{2}}})$$ (4)

**Formula of the min-max accuracy**

$$\begin{array}{c} \text{Min-Max}\;\text{Accuracy}=\text{mean}(\frac{\text{min}(y,x)}{\text{max}(y,x)}) \end{array}$$ (5)

In the above formulas, *x* represents the predicted Pn, and *y* represents the actual Pn. The evaluation metrics provide useful information to decide whether the model is a good fit, especially since the given dataset in this paper is skewed and small sized.

## Results and discussion

### Predictive analytics

We selected four regression methods that are suitable to work with the dataset given in this study. The first method implemented in this study is the eXtreme Gradient Boosting (XGBoost) model, which was proposed by Tianqi Chen as an implementation of gradient boosting machines [12]. XGBoost is insensitive to descriptive features that are highly correlated with response variables and is extremely flexible to work with in almost any regression or classification problems. The support vector machine (SVM) algorithm is a supervised machine learning algorithm implemented in this study as a regression solution [13]. The SVM algorithm’s ability to address highly correlated variables for regression makes this approach suitable for this study. Random forest (RF), invented by Leo Breiman, is a machine learning algorithm for both classification and regression tasks [14]. RF’s ability to deal with overfitting, ability to measure easily the importance of features, and insensitivity to correlated variables makes it another suitable algorithm for this study. The final selected algorithm, the generalized additive model (GAM), proposed by Trevor Hastie, is a hybrid algorithm to address effectively both linear and nonlinear regression problems [15]. GAM is a typical statistical model representing contemporary linear and nonlinear modeling algorithms. GAM is used as a benchmark regression model to predict the Pn as a comparison to XGBoost, SVM, and RF. Additionally, the cross-validation and regularization mechanisms were implemented in XGBoost, SVM, RF, and GAM to ensure the validity of the outputs. Tables 4–7 lists the results generated from XGBoost, SVM, RF, and GAM.

**Table 4:** Evaluation metrics—XGBoost.

Method	COR	RMSE	MAE	R^2^	Min-Max ACC	SD
XGBoost—Testing	0.77	2.57	1.12	0.60	0.93	3.97
XGBoost—Training	0.99	0.02	0.02	0.99	0.99	3.97

**Table 5:** Evaluation metrics—SVM.

Method	COR	RMSE	MAE	R^2^	Min-Max ACC	SD
SVM—Testing	0.73	2.77	1.31	0.53	0.92	3.97
SVM—Training	0.99	0.20	0.19	0.99	0.99	3.97

**Table 6:** Evaluation metrics—RF.

Method	COR	RMSE	MAE	R^2^	Min-Max ACC	SD
RF—Testing	0.69	2.99	2.13	0.47	0.87	3.97
RF—Training	0.97	1.33	0.96	0.94	0.93	3.97

**Table 7:** Evaluation metrics—GAM.

Method	COR	RMSE	MAE	R^2^	Min-Max ACC	SD
GAM—Testing	0.33	3.96	2.94	0.11	0.82	3.97
GAM—Training	0.76	2.59	1.95	0.57	0.87	3.97

The four selected predictive models can be classified into two categories: tree-based and non-tree-based models. The tree-based predictive models include XGBoost, SVM, and RF. Therefore, GAM belongs to the non-tree-based category. Nonparametric tree-based models are naturally immune from the multicollinearity issue. Therefore, the GAM algorithm, like other non-tree-based predictive models, can be impacted by the multicollinearity issue. The purpose of these models is to use leaf shape data to predict the Pn with a high accuracy.

### Extreme gradient boosting

We implemented a 10-fold cross-validation mechanism with a grid search data frame in the extreme gradient boosting (XGBoost) model. This grid search data frame is then embedded and fine-tuned in the train function from the R caret package. The key parameters include the number of trees (nrounds), maximum tree depth (max_depth), learning rate (eta), regularization (gamma), column sampling (colsample_bytree), minimum leaf weight (min_child_weight), and row sampling (subsample), which are fine-tuned through the grid search with the 10-fold cross-validation design. The final fine-tuned parameters nrounds, max_depth, eta, gamma, colsample_bytree, min_child_weight, and subsample are set to 100, 10, 0.2, 0, 0.3, 1 and 0.9, respectively.

The Pn predicted by fine-tuned XGBoost model has a correlation of 0.77, RMSE of 2.57, MAE of 1.12, R^2^ of 0.60, and min-max accuracy of 0.93 with the actual Pn in testing. Moreover, the correlation, RMSE, MAE, R^2^, SD, and min-max accuracy in training are 0.99, 0.02, 0.02, 0.99, and 0.99, respectively, between predicted Pn and actual Pn (refer to Table 4).

**Fig 7:**
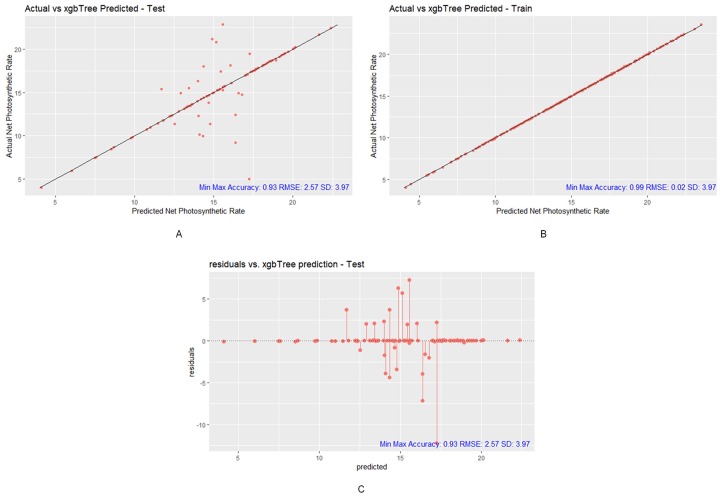
Prediction results of XGBoost model. (A) XGBoost testing plot with Min-Max Accuracy 0.93, RMSE 2.57 and SD 3.97, (B) XGBoost training plot with Min-Max Accuracy 0.99, RMSE 0.02 and SD 3.97, (C) XGBoost test residual plot with Min-Max Accuracy 0.93, RMSE 2.57 and SD 3.97 (R package ggplot2).

By comparing the RMSE scores of the XGBoost testing and training predictions, we can observe that there are some overfit models (refer to Fig 7A and 7B). However, the XGBoost model performed the best among all the models overall. The correlation between the actual Pn and the predicted Pn is 0.77 in testing, which is a considerably high number. This finding means that the XGBoost-generated predictions are highly correlated with the observed Pn values. In addition, the min-max accuracy between actual Pn and predicted Pn is an extremely high number: 0.93 in testing. The perfect min-max accuracy score is 1, which means that the predicted Pn values are incredibly close to the ideal predictions.

To further verify the goodness of fit of the XGBoost model in testing, we created a residual plot to visualize how the errors are distributed above and under the 0-residual line (refer to Fig 7C). The XGBoost residual plot shows that the residuals fall in a symmetrical pattern towards the middle of the plot. This finding means that the XGBoost model reasonably fits the data to predict the Pn values with high correlation, low RMSE, low MAE, moderate R^2^, and a very high min-max accuracy score. Using the RMSE vs. SD approach, when a model’s RMSE is smaller the than SD, then that model generates better predictions that by using the mean. In this study, the XGBoost model’s RMSE score of 2.57 is much lower than the SD of 3.97, which means that the XGBoost model’s predictions are much more accurate than the mean of the actual Pn values to represent the Pn. Those findings demonstrate that the XGBoost model is a good fit and capable of making accurate predictions when the predicted results are strongly and closely associated with the actual Pn values.

### Support vector machine

We fine-tuned the support vector machine (SVM) model using the R e1071 package with a 10-fold cross-validation design. The model parameters include gamma, cost and epsilon, which were fine-tuned using the tune.svm function. The best tuned parameters are 30, 5, and 0.05 for gamma, cost, and epsilon, respectively, with a cross-validation design of 10. The SVM model was then trained with the radial kernel.

The SVM model predicted that the Pn has a correlation of 0.73, RMSE of 2.77, MAE of 1.31, R^2^ of 0.53, and min-max accuracy of 0.92 to the actual Pn in testing. Moreover, the correlation, RMSE, MAE, R^2^, SD, and mi- max accuracy in training are 0.99, 0.20, 0.19, 0.99, and 0.99, respectively, between the predicted Pn and actual Pn (refer to Table 5).

**Fig 8:**
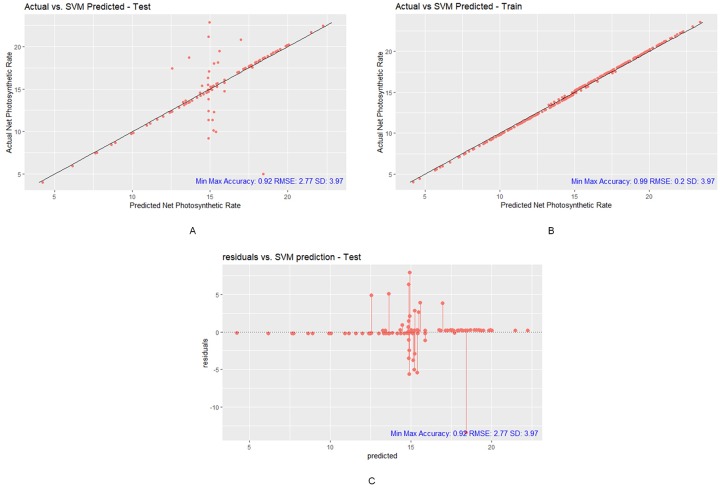
Prediction results of SVM model. (A) SVM testing plot with Min-Max Accuracy 0.92, RMSE 2.77 and SD 3.97, (B) SVM training plot with Min-Max Accuracy 0.99, RMSE 0.2 and SD 3.97, (C) SVM test residual plot with Min-Max Accuracy 0.92, RMSE 2.77 and SD 3.97 (R package ggplot2).

We can observe that the SVM model, during testing, 1) the predicted Pn value a correlation of 0.73 with the actual Pn values, 2) the RMSE of 2.77 is much smaller than the SD of 3.97, 3) has a small MAE (1.31), 4) yields a moderate R^2^ between the actual and predicted Pn values, and 5) has a high min-max accuracy score of 0.92 (refer to Table 5, Fig 8A and 8B). This finding means that the trained SVM is capable of making Pn predictions with reasonably high accuracy.

Furthermore, the SVM residual plot illustrates that the residual plot does follow a symmetrical pattern above and below the 0-residual line towards the middle of the plot (refer to Fig 8C). Based on the evaluation metrics and the residual plot, the SVM model is a good fit to predict the Pn value with a high accuracy and low error using the noisy and skewed data of this study.

### Random forest

The random forest (RF) model was fine-tuned in two steps. The first step was to generate the optimal number of trees. A grid search data frame was created to contain the different numbers of trees ranging from 50 trees to 4800 trees. Then, the tree grid search design was tuned using the train function from the R caret package. The fine-tuned result for the optimal number of trees is 300. The second step was to fine-tune the mtry parameter. The mtry parameter stands for the number of variables to split at each tree node, which is another very important tunable parameter in an RF model. The tuneRF function from the R randomForest package was used, and the best mtry value was calculated as 4.

The RF model-predicted Pn has a correlation of 0.69, RMSE of 2.99, MAE of 2.13, R^2^ of 0.47, and min-max accuracy of 0.87 to the actual Pn in testing. Moreover, the correlation, RMSE, MAE, R^2^, SD, and min-max accuracy in training are 0.97, 1.33, 0.96, 0.94, and 0.93, respectively, between predicted Pn and actual Pn (refer to Table 6). The first impression of this model is that the RMSE is smaller than the SD in testing. This finding means that the RF model is capable of predicting some moderately accurate Pn results. In addition, the min-max accuracy is 0.87, which is a relatively high score, which means that the average distance of predicted Pn values is somewhat close to the actual Pn values.

**Fig 9:**
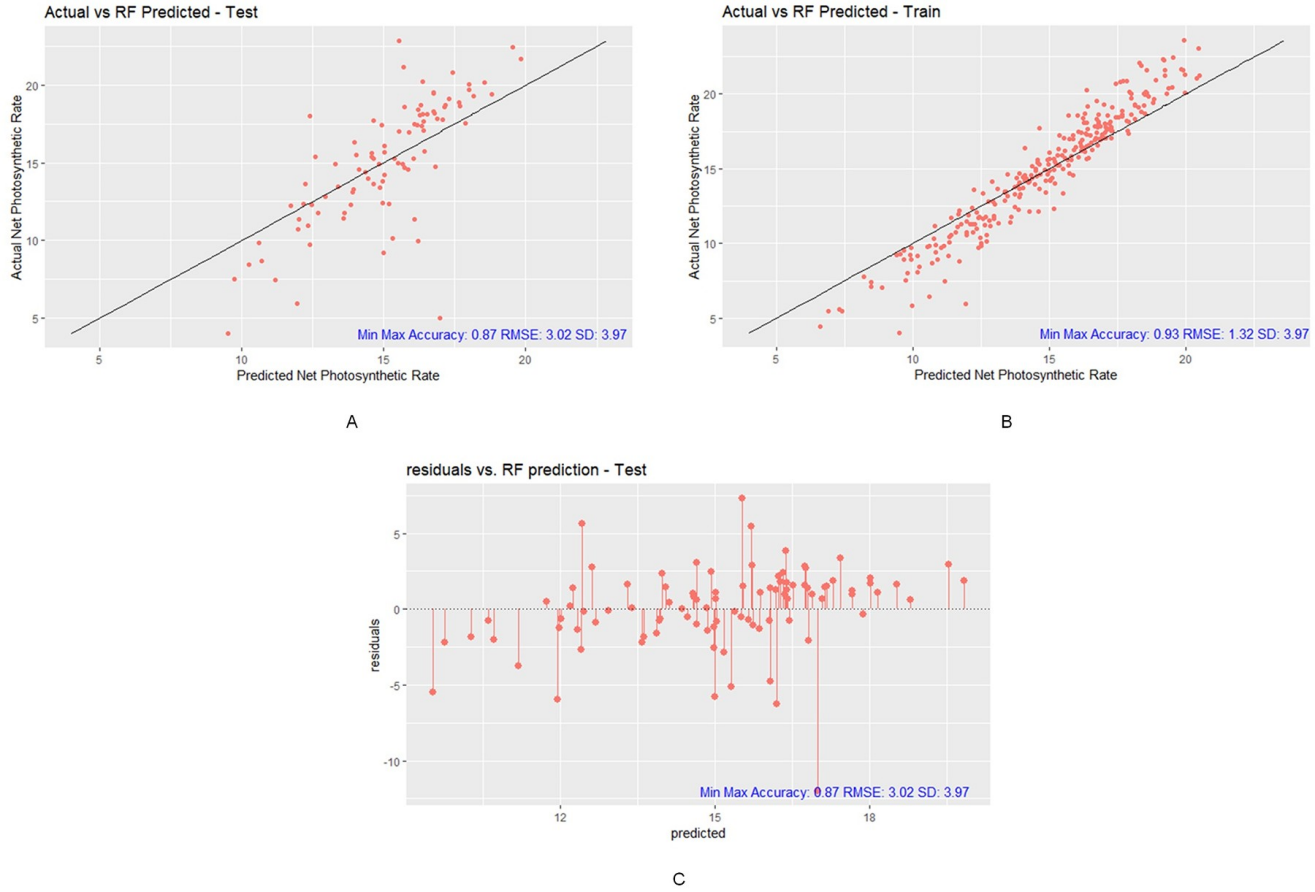
Prediction results of RF model. (A) RF testing plot with Min-Max Accuracy 0.87, RMSE 3.02 and SD 3.97, (B) RF training plot with Min-Max Accuracy 0.93, RMSE 1.32 and SD 3.97, (C) RF test residual plot with Min-Max Accuracy 0.87, RMSE 3.02 and SD 3.97 (R package ggplot2).

The next step is to verify how good the RF model fits the actual Pn data. According to Fig 9A and 9B, we can observe that both the predicted Pn values from testing and training follow the 45-degree line of best fit. However, from the residual plot from testing (refer to Fig 9C), the beginning quarter and the ending quarter of the plot are not distributed symmetrically. The middle half of the residual plot follows the symmetrical pattern. Overall, the RF model could yield a moderately accurate prediction with a reasonably good fit.

### Generalized additive model

The GAM is impacted by highly correlated variables. To avoid the impact of multicollinearity, a redundant variable removal algorithm was implemented by evaluating the correlation of the descriptive features with a cutoff value of 0.70. After the remove of redundant variables, the remaining descriptive features are ratio and factor. To add predictive power of the model, the cluster variable is added in the GAM as a uncorrelated descriptive feature. A grid search mechanism is implemented in the GAM fine-tuning process with parameter knots for ration and factor, sp, and gamma.

The GAM-predicted Pn has a correlation of 0.33, RMSE of 3.96, MAE of 2.94, R^2^ of 0.11, and min-max accuracy of 0.82 to the actual Pn in testing. Moreover, the correlation, RMSE, MAE, R^2^, SD, and min-max accuracy in training are 0.76, 2.59, 1.95, 0.57, and 0.87, respectively, between predicted Pn and actual Pn (refer to Table 7).

**Fig 10:**
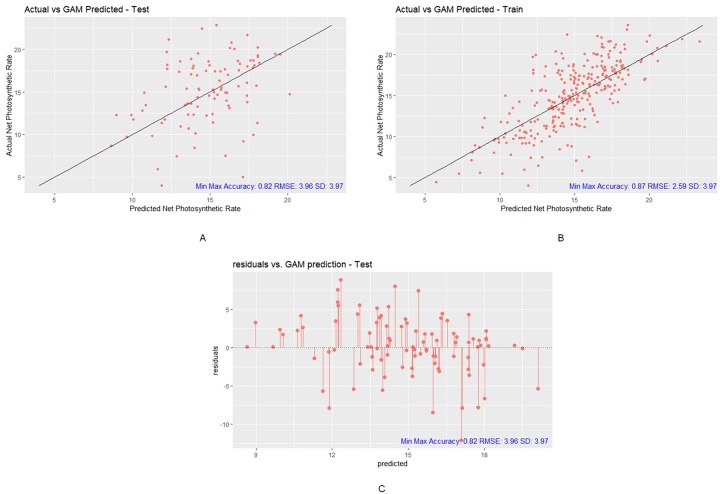
Prediction results of GAM model. (A) GAM testing plot with Min-Max Accuracy 0.82, RMSE 3.96 and SD 3.97, (B) GAM training plot with-Min Max Accuracy 0.87, RMSE 2.59 and SD 3.97, (C) GAM test residual plot with Min-Max Accuracy 0.82, RMSE 3.96 and SD 3.97 (R package ggplot2).

We observed that the RMSE of 3.96 is smaller than the SD of 3.97 in testing (refer to Fig 10A and 10B), which means that the GAM’s prediction is slightly better than using the mean Pn to represent the Pn predictions. There is no overfitting in this model. However, the correlation of 0.33 and R^2^ of 0.11 are relatively small. This means that the association between the actual and predicted Pn values is relatively weak. Moreover, the MAE is 2.94, which can be considered low. Overall, from the evaluation metrics, the GAM is capable of making reasonable, but weak, Pn predictions using the given small, noisy and skewed leaf phenotype data.

In addition to the GAM, the first quarter and the last quarter did not follow a symmetrical pattern. The middle half of the residual plot did follow a symmetrical pattern (refer to Fig 10C). The good fit of GAM is somewhat acceptable.

## Conclusions

According to our evaluation metrics (refer to Table 8), the XGBoost model performed the best. The correlation, R^2^, and min-max accuracy are the highest among the four proposed prediction methods. The RMSE and MAE are also the lowest. Additionally, according to the residual plot, the residuals of the XGBoost-predicted Pn values followed a random pattern towards the middle of the plot, and the random pattern above and below the 0-residual line made the XGBoost a good fitting model.

**Table 8:** Evaluation metrics—XGBoost, SVM, RF, and GAM.

Evaluation metrics—XGBoost, SVM, RF, and GAM							
Method	COR	RMSE	MAE	R^2^	Min-Max ACC	SD	Decision
XGBoost—Testing	0.77	2.57	1.12	0.6	0.93	3.97	Yes
SVM—Testing	0.73	2.77	1.31	0.53	0.92	3.97	No
RF—Testing	0.69	2.99	2.13	0.47	0.87	3.97	No
GAM—Testing	0.33	3.96	2.94	0.11	0.82	3.97	No

As a result, we recommend XGBoost to make accurate and reliable Pn predictions using small, noisy, and skewed leaf phenotype data. This study verified that it is possible to use leaf phenotype data to predict the Pn exactly, effectively, and economically. Precise and economic algorithms to predict the Pn can provide strong evidence to manage and monitor the photosynthesis process and CO_2_ emissions efficiently and effectively worldwide.

### Future works

Four future works can be implemented in this study to enhance the Pn prediction power. The following are some ideas that could benefit future works:

1. More leaf phenotype and photosynthesis data can be collected in the future to provide more insights regarding the association between leaf phenotype traits and the Pn to build a more robust predictive model.
2. A deep learning regression model can be implemented in predicting the Pn when a large dataset is available for such studies because the deep learning algorithms have been proven to be superior to many other prediction algorithms in many different industries.
3. More predictors can be introduced into the Pn predictive model to enhance the prediction reliability. These new predictors, such as leaf color and leaf hyperspectral data, should not have a strong impact on the environmental and geographical conditions.
4. More tree species data are recommended to be implemented in the Pn prediction model to improve the model’s generalizability to other trees or plants.

The big data era is here. Massive amounts of data are being generated and stored by companies, organizations, and research institutes. The same is happening in the forestry and ecology industries every day. We shall utilize our predictive and analytical skills as data scientists to make more robust and precise predictions to protect our earth.

## Supporting information

S1 Dataset Dataset of *P*. *simonii* leaf phenotype and photosynthesis. (CSV)
